# Obesity and Dental Decay: Inference on the Role of Dietary Sugar

**DOI:** 10.1371/journal.pone.0074461

**Published:** 2013-10-10

**Authors:** J. Max Goodson, Mary Tavares, Xiaoshan Wang, Richard Niederman, Maryann Cugini, Hatice Hasturk, Roula Barake, Osama Alsmadi, Sabiha Al-Mutawa, Jitendra Ariga, Pramod Soparkar, Jawad Behbehani, Kazem Behbehani

**Affiliations:** 1 Applied Oral Sciences, the Forsyth Institute, Cambridge, Massachusetts, United States of America; 2 Nutritional Services, Dasman Diabetes Institute, Kuwait City, Kuwait; 3 Biomedical Research, Dasman Diabetes Institute, Kuwait City, Kuwait; 4 Oral Health, Ministry of Health, Kuwait City, Kuwait; 5 School Oral Health Program, Ministry of Health, Kuwait City, Kuwait; 6 Applied Oral Sciences, the Forsyth Institute, Cambridge, Massachusetts, United States of America; 7 Administration, Faculty of Dentistry, Kuwait University, Kuwait City, Kuwait; 8 Administration, Dasman Diabetes Institute, Kuwait City, Kuwait; University Hospital of the Albert-Ludwigs-University Freiburg, Germany

## Abstract

**Objective:**

To evaluate the relationship of children’s obesity and dental decay.

**Methods:**

We measured parameters related to obesity and dental decay in 8,275 4^th^ and 5^th^ grade Kuwaiti children (average age = 11.36 years) in a cross-sectional study. First to determine body weight, height, age for computation of BMI . Second, to determine numbers of teeth, numbers of fillings and numbers of untreated decayed teeth to determine extent and severity of dental disease. From these measurements, we computed measures of dental decay in children from four body weight categories; obese, overweight, normal healthy weight and underweight children.

**Results:**

The percentage of children with decayed or filled teeth varied inversely with the body weight category. The percentage of decayed or filled teeth decreased from 15.61% (n=193) in underweight children, to 13.03% (n=4,094) in normal healthy weight children, to 9.73% (n=1,786) in overweight children to 7.87% (n=2,202) in obese children. Differences between all groups were statistically significant. Male children in this population had more dental decay than female children but the reduction of tooth decay as a function of BMI was greater in male children.

**Conclusions:**

The finding of an inverse obesity-dental decay relationship contradicts the obesity-sugar and the obesity-dental decay relationship hypotheses. Sugar is well recognized as necessary and sufficient for dental decay. Sugar is also hypothesized to be a leading co-factor in obesity. If the later hypothesis is true, one would expect dental decay to increase with obesity. This was not found. The reasons for this inverse relationship are not currently clear.

## Introduction

The findings of two systematic reviews with meta-analysis associate obesity with sugar intake [1] with dental decay[2]. Increased dental caries has long been associated with increased sugar consumption [3,4]. Dental caries does not develop in the absence of sugar [5]. Thus, dental caries has become a surrogate marker for sugar intake.

The current controversy relating obesity to sugar consumption has migrated to the level of affecting public policy despite the weakness of supporting data. A recent systematic review[1] concluded that unrestricted intake sugar experimentally increased body weight by only 0.8 kg. It was also concluded that studies involving isocaloric substitution of other carbohydrates for sugar had no effect on body weight. These data can hardly be considered evidence that obesity is caused by sugar consumption.

With this conundrum in mind, we have chosen to focus on the dental decay patterns of obese children. Dental caries clearly has a multifactorial etiology. There are food characteristics (tooth adherence), individual factors ( plaque microbial composition, saliva characteristics and tooth structure differences) behavioral factors (frequency of sugar intake, oral hygiene, dental care) and socioeconomic factors (parental education, parental income). Despite this complexity, however, dental caries, has been particularly associated with sugar from the time of our earliest archeological findings[3] to our latest scientific research [4]. Although the advent of water fluoridation may have reduced the disease severity in some populations, the association between fermentable carbohydrate consumption and dental decay is generally acknowledged[6]. The extent to which this association exists was recognized by the United States’ National Research council in observing that ‘dental caries does not develop in the absence of fermentable carbohydrates [5].

The current report derives from a larger longitudinal study evaluating the development, causes, and prevention of obesity in Kuwaiti children. The size was selected to provide approximately 500 children with metabolic syndrome (estimated 5%). In this population obesity is prevalent [7-9], drinking water naturally contains 0.5-1.5 ppm fluoride [10] and is not artificially fluoridated. 

The aim of this work was to challenge the belief that obesity is commonly caused by increased sugar consumption. Although difficult to prove disassociation, the indelible mark of dental decay does not support association of sugar consumption with obesity in children.

## Materials and Methods

Between October 2, 2011 and May 15 of 2012 data was collected from 8,319 children during 182 visits to 39 Kuwaiti schools with approximately equal distribution to all governorates of Kuwait. These schools serve all social levels of native Kuwaiti children but not expatriate children. In total, there were 52,687 children in 250 primary schools from which to select. Schools were chosen by the Director of the School Oral Health Program in Kuwait (J,A.) based on an expressed willingness of the school administration to allow their children to participate in our clinical trial. 8,275 children had complete oral and body weight measurements suitable for evaluation in this analysis. Children in the study were in either the 4 ^th^ or 5^th^ grades with written parental informed consent and verbal child assent obtained prior to participation. All volunteers were accepted but the selection was not randomized across the population. Written consent forms were collected and filed for future reference. The study was reviewed and approved by the Forsyth Institutional Review Board and the Dasman Institute Human Ethical Review committees (RA/065/2011 and RA/005/2011).

### Clinical Examination

Children of this age have a mixed primary and permanent dentition. Oral examinations were conducted by licensed dentists assisted by trained nurses using portable dental chairs, halogen lights and intraoral mirrors. Examiners received training in diagnosis of visible decay and gingival health prior to conducting examinations by a calibrated examiner. No radiographic images were taken and no dental explorers were used. Numbers of primary (deciduous) and permanent teeth present were recorded as well as the number of teeth with fillings and visible unfilled decay and entered with age and identification into tablet computers (iPad, Apple Corporation, Cupertino CA) for later downloading to a database. Height was measured by using a stadiometer in centimeters and weight was measured using a digital scale in kilograms. .

### Data Analysis

Body weight categories were defined using WHO growth reference data for 5-19years [11]. By WHO recommended cut-off values, obese was defined as a Z-score greater than 2 SD, overweight was defined as greater than 1 SD, normal healthy weight was defined as between1 and -2 SD. Underweight was defined as <-2 SD.. .

Since no radiographs were taken, missing teeth could not be evaluated. For this reason, the percent of decayed or filled teeth were used as a measure of dental caries severity. To provide this measure in children with mixed dentition (having both primary and permanent teeth) the outcome variable, percent of decayed or filled teeth, was computed as follows:

Decayed or filled teeth(%)=100 x [(number of primary teeth with fillings)+(number of permanent teeth with fillings) +(number of decayed primary teeth)+(number decayed permanent teeth)]/[(number of primary teeth)+(number of permanent teeth)].

 These data were clearly not normally distributed so that conventional parametric analysis could introduce bias. Two distributions designed to fit fractionalized data were tested ( zero –inflated beta regression[12] and quasi-likelihood with logit link[13]) and found inadequate descriptors of these data. To account for the non-normality of fraction outcome, a multivariate rank-based Wilcoxon regression method [14] was applied to fit the proportion of decayed or filled teeth. In essence, for a model*y*_*i*_=*β*_0_+*X*_*i*_ *β*+*ϵ*_*i*_, the Wilcoxon estimate of *β*is defined to minimize the dispersion

$$D(\beta )=\sum_{i=1}^{n}\left(R(\varepsilon_{i}\right)-\frac{n+1}{2})\varepsilon_{i}$$

where *R*(*ϵ_i_*)is the rank of errors. Age (years) and gender (1=male, 0=female) were adjusted when evaluating the relationship between the proportion of decayed or filled teeth and body weight categories or body mass index (BMI, mass(kg)/height(m)^2^).

Enumerated data such as the number of children with untreated decay were tested for significant differences by Pearson chi-square analysis. Mean values are listed with ± standard deviation unless otherwise noted.

## Results

In this group of 8,275 children, the average age was 11.36 ± 0.1 years, with a mixed dentition of primary (n=6.6 ± 4.0 teeth/child) and permanent teeth (n= 16.7 ± 4.8 teeth/child). The total number of teeth in each child (primary + permanent) was relatively constant (n=23.3 ± 2.0 teeth/child). The population was 38.3% male.

### Prevalence of dental decay

**Figure 1:**
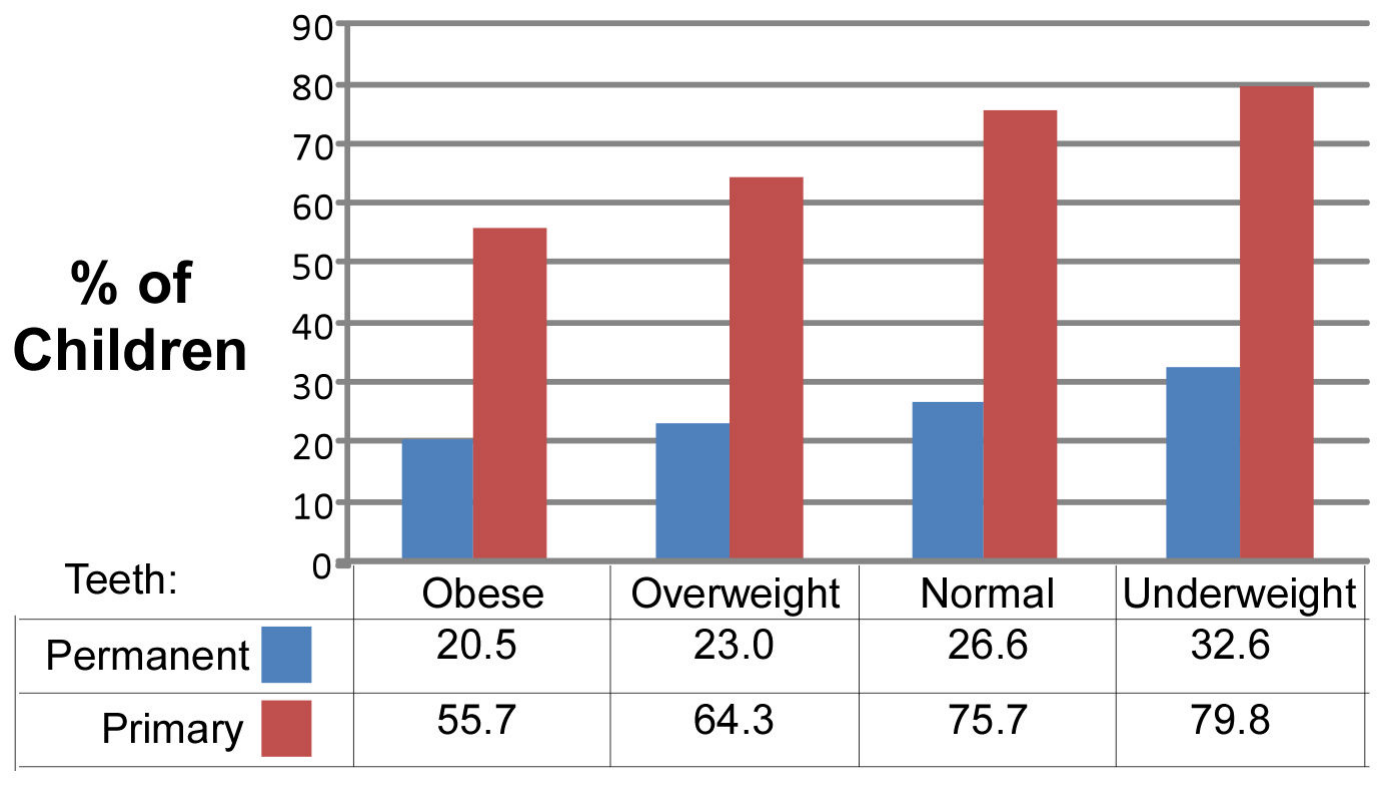
Prevalence of dental decay. The percent of children in each body weight category with at least 1 decayed or filled tooth (computed separately for permanent and primary teeth)..

The percent of children with dental decay in permanent teeth varied from 20.5% in obese children to 32.6% in underweight children (Figure 1). The percent of children with dental decay in primary teeth varied from 55.7% in obese children to 79.8% in underweight children. The trend toward reduced prevalence of dental decay in obese children was highly significant considering either permanent or primary teeth.

### Severity of Decay

**Figure 2:**
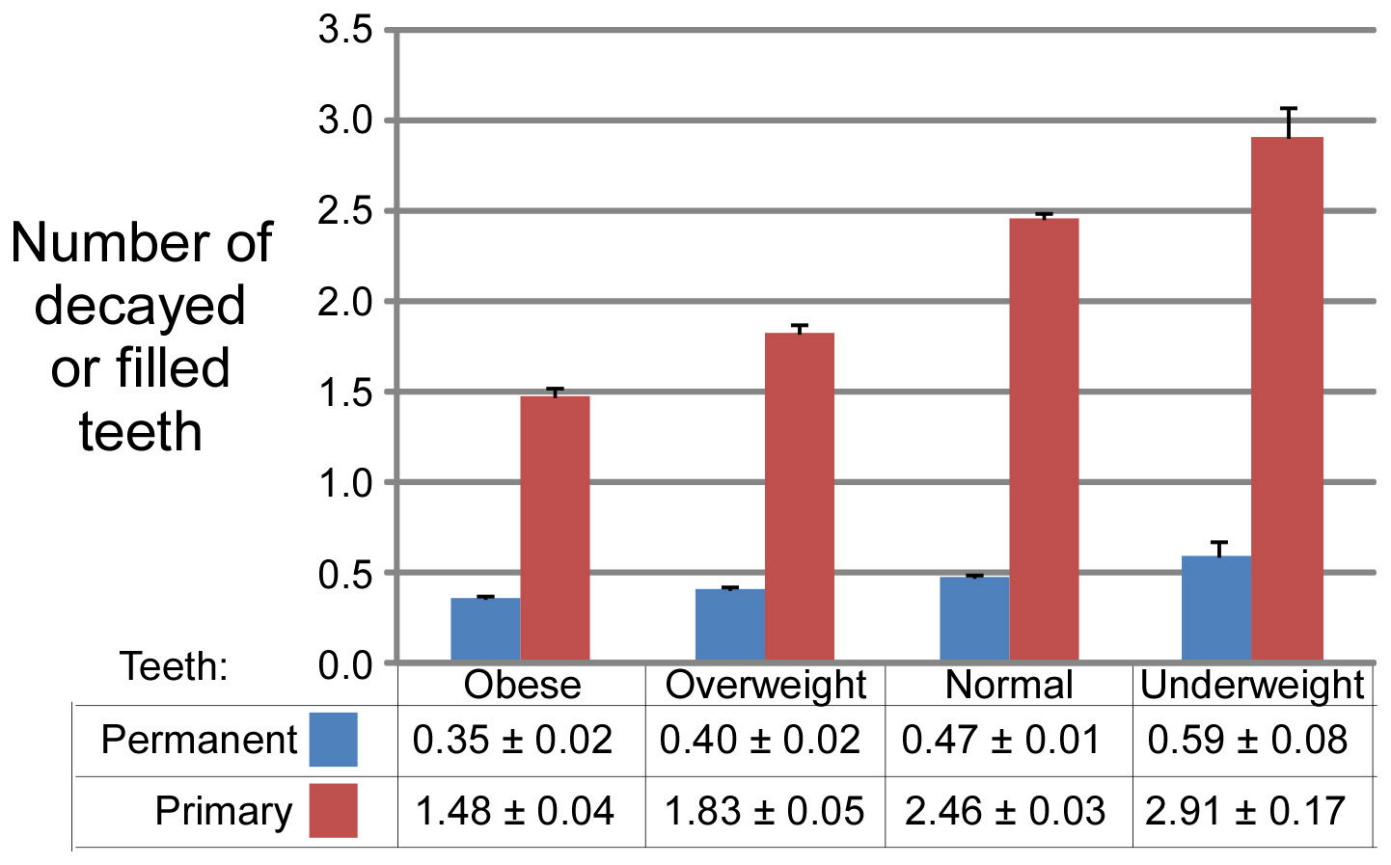
Severity of dental decay measured as mean number of decayed or filled teeth in both permanent and primary dentition of children (mean ± S.E.M.) of different body weight categories.

The severity of dental decay measured as the average number of decayed or filled teeth also decreased with increasing body weight category (Figure 2). The rate of dental caries reduction with increasing body weight was less in permanent teeth than in primary teeth, but both changes were highly significant.

### Percent of teeth decayed or filled

**Figure 3:**
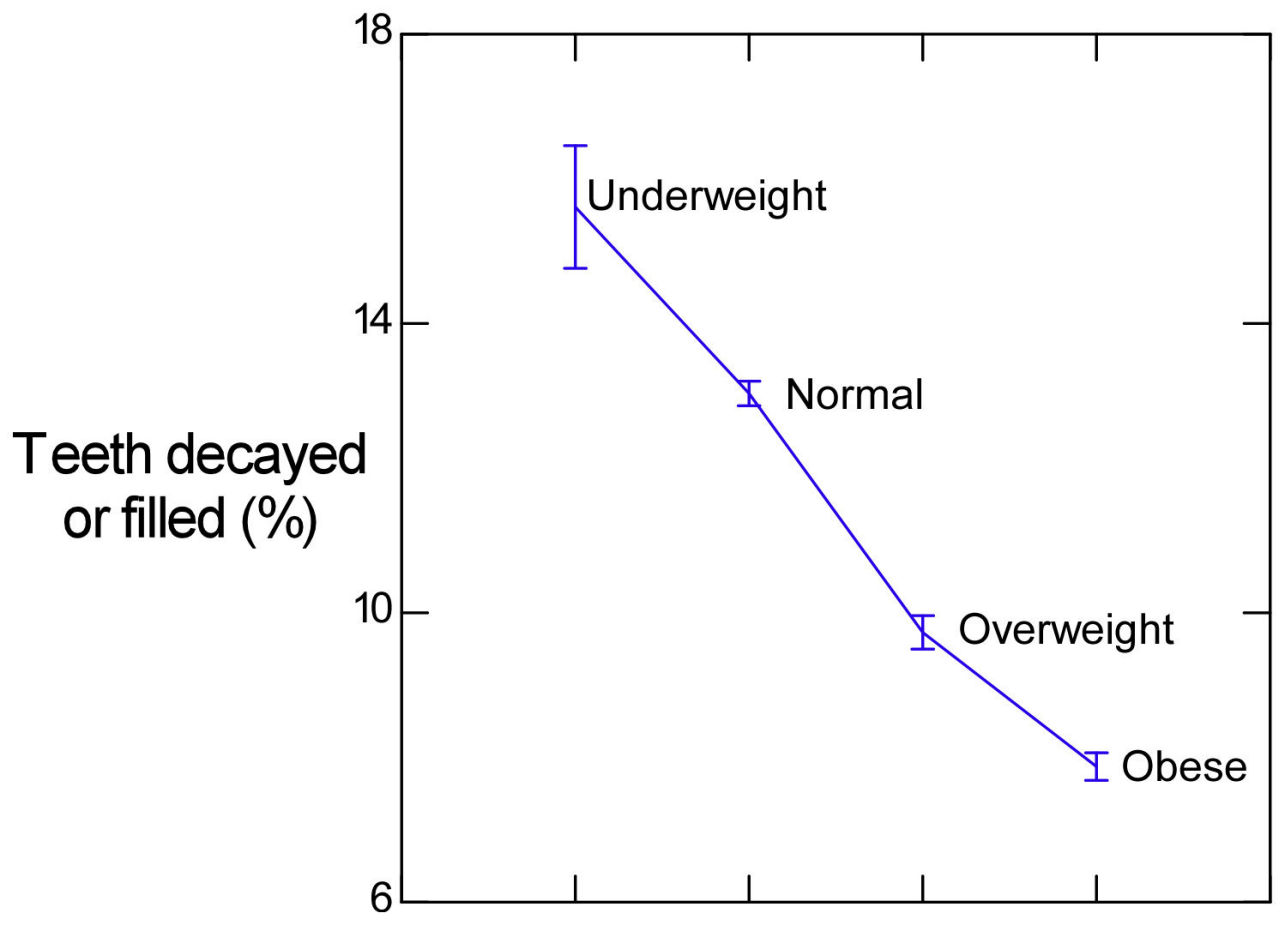
Extent of dental decay (including both primary and permanent teeth) by body weight category (male and female children combined). Plotted values are means and whiskers are SEM.

When dental decay of primary and permanent teeth were combined as an extent of decay measure (Table 1), male children had more dental decay than female in all body weight categories, but both exhibited reduced dental decay with an increase in body weight category. Dental decay is seen to decrease as body weight increases (Table 1, Figure 3). From these data, obese children had approximately half the number of decayed or filled teeth seen in underweight or normal healthy weight children.

**Table 1:** Percent of teeth decayed or filled by body weight category and age for all children and for male and female children separately.

	All Children			Male	Female
Body weight category	Decayed or filled teeth [% ± SEM]	Age [Years ± SEM]	N	Decayed or filled teeth [% ± SEM (N)]	Decayed or filled teeth [% ± SEM (N)]
Underweight	15.61 ± 0.85	11.53 ± 0.04	193	19.91 ± 1.55 (64)	13.48 ± 0.96 (129)
Normal	13.03 ± 0.17	11.38 ± 0.02	4094	14.34 ± 0.29 (1489)	12.29 ± 0.21 (2605)
Overweight	9.73 ± 0.23	11.36 ± 0.01	1786	10.66 ± 0.41 (615)	9.24 ± 0.28 (1171)
Obese	7.87 ± 0.19	11.29 ± 0.01	2202	8.12 ± 0.28 (1002)	7.67 ± 0.26 (1200)
All categories	11.01 ± 0.11	11.36 ± 0.01	8275	11.76 ± 0.19 (3170)	10.53 ± 0.14 (5105)

Analysis of these data by Wilcoxin rank regression (Table 2) reveals that age, gender, body weight category and age all significantly contributed to tooth decay. By this method, after adjusting for age and gender, overweight children would on average have 3.51% fewer decayed or filled teeth than those who are normal healthy weight. Obese children would average 4.64% fewer than normal weight children and underweight children, would average 2.23% more decayed or filled teeth than normal weight children. All differences tested were highly significant.

**Table 2:** Analysis by Wilcoxin rank regression to evaluate the hypothesis that numbers of teeth decayed or filled corrected for age and gender were significantly related to body weight category.

		SE	t	p
Intercept	15.09	0.81	18.71	<0.001
Age	-0.37	0.06	-6.33	<0.001
Gender	0.95	0.12	7.68	<0.001
Overweight	-3.51	0.15	-22.77	<0.001
Obese	-4.64	0.14	-32.17	<0.001
Underweight	2.23	0.44	5.05	<0.001

β values were computed relative to normal healthy weight children.

**Figure 4:**
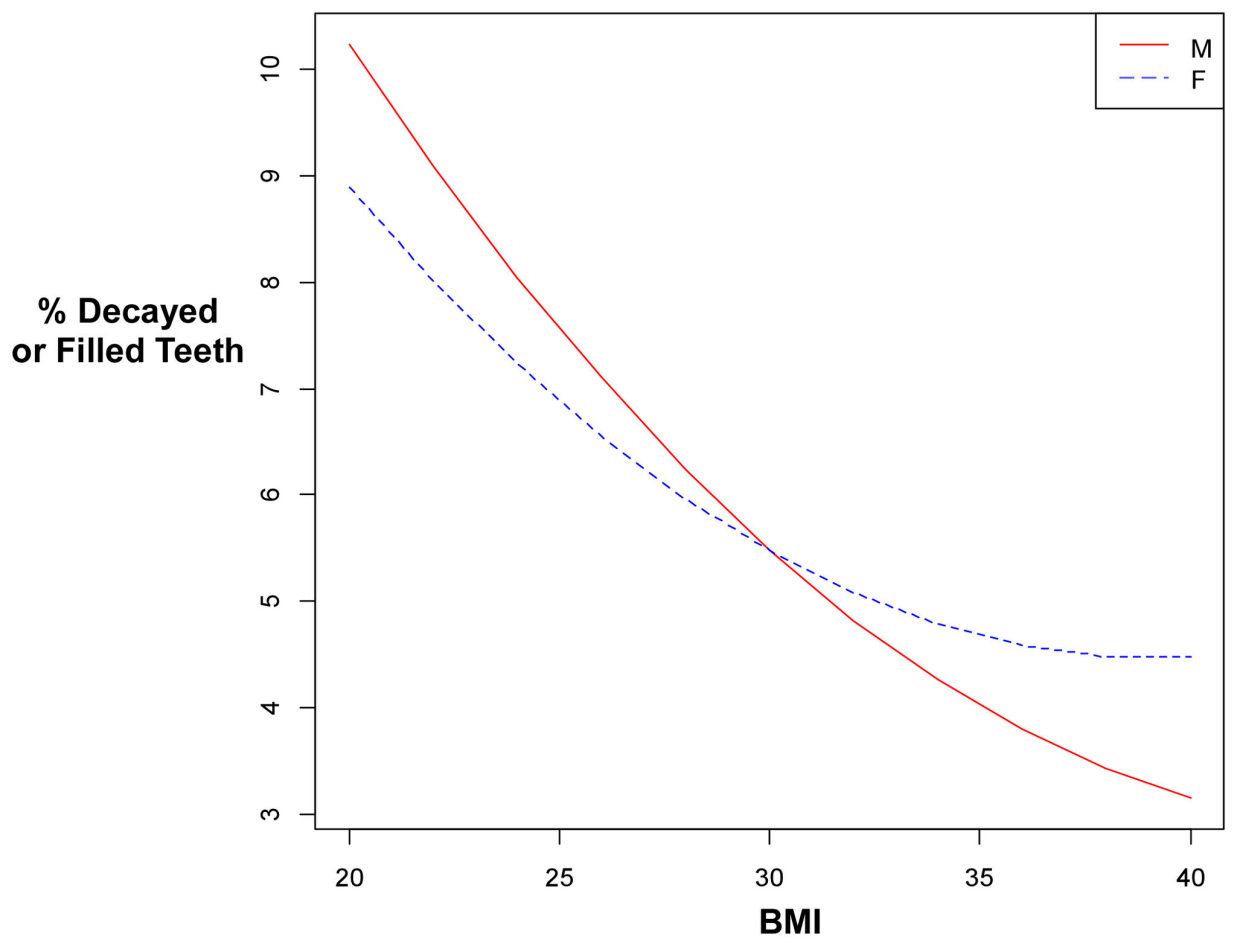
Relationship between BMI and gender for boys and girls indicating that with obesity (BMI>30), dental decay was reduced to a greater extent in boys than in girls. Values were computed from the regression model described in table 3.

Analysis of the percent of teeth decayed or filled related to BMI appear in table 3. After adjusting for age and gender, the number of teeth decayed or filled decreased by approximately by 0.95% for each BMI interval. Significance in the BMI2 term indicates that the association of BMI and the percent of teeth decayed or filled were nonlinear. Significance in the BMIxGender interaction term indicates that the effect of BMI was different between genders (Figure 4). All differences tested were highly significant. The interaction between gender and BMI indicated that boys had less dental decay than in girls, particularly when BMI exceeded 30

**Table 3:** Analysis by Wilcoxin rank regression to evaluate the hypothesis that numbers of teeth decayed or filled corrected for age and gender was significantly related to BMI.

		SE	t	p
Intercept	25.98	1.47	17.63	<0.001
Age	-0.29	0.10	-3.01	0.003
Gender	4.11	0.82	5.03	<0.001
BMI	-0.95	0.08	-11.98	<0.001
BMI^2^	0.01	0.00	8.02	<0.001
BMIxGender	-0.14	0.04	-3.65	<0.001

## Discussion

The population in Kuwait is 80% Arab. The children of this study all attended public schools which enrolls only native Kuwaiti children. The socio-economic level of these children is close to the highest in the world since per capita income in Kuwait is high. The diet of Kuwaiti children is in many ways similar to that of the United States and is greatly affected by the same ubiquitous fast food establishments.

In this population, a reduction in dental decay with increasing obesity was observed both in measures of prevalence and severity. This suggests that fermentable carbohydrate consumption may not be a primary cause of obesity in Kuwaiti children. This may also be true of other populations. A recent systematic review of studies relating obesity and dental caries identified 14 publications between 1980 and 2010 that contribute to the question [2]. Adding our work to this list results in the data of table 4.

**Table 4:** Published studies that relate dental caries and obesity[2]*.

										
	Author	Country	Age (yr)	N	Conclusion	Pop. Sums	Ave size		Quality	Ref
	Alm et al.	Sweden	13.5-16.4	402	1	5,655	1,131		8	33
	Sharma & Heyde	India	8-12	500	1				6	36
	Vazquez-Nava et al.	Mexico	4-5	1160	1				9	16
	Willershausen et al.	Germany	6-11	1290	1				5	37
	Gerdin et al.	Sweden	4-10	2303	1				11	35
	Sheller et al.	USA	2-5	293	0	9,919	1,984		7	57
	Tramini et al.	France	12	835	0				8	58
	Sadeghi & Alzdeh	Iran	6-11	1007	0				4	45
	Granville-Garcia et al.	Brazil	1-5	2651	0				4	39
	Chen et al.	Korea	3	5133	0				6	38
	Narksawat et al.	Thailand	12-14	862	-1	25,340	5,068		5	44
	Oliveira et al.	Brazil	1-5	1018	-1				10	59
	Kopycka-Kedzierawski et al.	USA	2-18	7568	-1				10	42
	Macek and Mitola	USA	2-17	7617	-1				10	43
	Goodson et al.	Kuwait	11	8275	-1					

*Reports are separated by conclusion (1 dental decay increases with obesity, 0 = there is no association between dental decay and obesity, - 1 = dental decay decreases with obesity). N=number of children, pop. Sums = the total number children studied with each conclusion, Ave. Size = the average size of a study with each conclusion.

As this table indicates, studies representing the conclusion that dental caries increases with obesity are much smaller and in the minority relative to those supporting the converse hypothesis. Based on the data of this study and majority of participants in recent credible published studies, it seems that dental caries may not increase and possibly decreases with obesity.

These observations raise questions that are difficult to answer. If obesity is specifically related to sugar consumption, why don’t obese children have more dental decay? How can dental decay be reduced in obese children if eating sugar causes their obesity? The diet of Kuwaiti children is high in fast food which is also associated with increased fat from fried foods and large portion size, either of which could account for their obesity. Clearly, considering the complexity of human research, we cannot account for all possible sources of interaction between the three variables sugar consumption, dental caries and obesity. That having been said, logic demands that we question the validity of the least well substantiated causal relationship, i.e. that sugar consumption is responsible for obesity.

We should also pause to consider the comparative validity of short-term controlled sugar feeding studies and 24-hour dietary recall with experiments of nature. Dental decay and sugar consumption are widely acknowledged as being effect and its cause. Many consider dental decay as a modulus of sugar consumption, particularly in unfluoridated communities such as Kuwait. Unquestionably, sugar consumption has pathogenic complications, not the least of which is dental decay. But evidence that sugar consumption is specifically related to obesity is much less compelling.

The results of this study raise yet another question. An assumption that sugar consumption causes or is primary to development of obesity would have been supported if dental decay increased with obesity. One might suggest a reason if no difference between dental decay and obesity was seen. So by what mechanism does dental decay decrease with increasing obesity? Studies that have reported decreasing dental decay with increasing obesity[15-18] have all concluded that “The relationship between overweight and dental caries in children is far more complex than can be explained by carbohydrate consumption alone.” The largest study[18] based on United States children concluded that “NHANES III and NHANES 1999-2002 provide no evidence to suggest that overweight children are at increased risk for dental caries”. This subject will clearly require more investigation. 

In the Kuwait study, only children in public schools were evaluated. This excludes all expatriate children and includes only native Kuwaiti. All native Kuwaiti children have free education, free medical care and free dental care. Dental care is dispensed from numerous clinics and is often available in the school clinic. Although there are income differences, all native Kuwaiti would be considered as having a relatively high socioeconomic status. Hence, there is no reason to assume that obese children had better dental care or that foreign populations biased the results of this study.

Dramatic changes have occurred in Kuwait over the last 50 years which undoubtedly affected both obesity and dental caries. Introduction of American fast food industry that followed the Gulf War (1990-1991), has likely affected eating habits. Introduction of a national preventative dental program for children [19] in 1982 has likely affected children’s oral health[20]. We acknowledge that these events could affect conclusions made from these data.

## Conclusions

Measurement of restored and decayed teeth in 8,275 eleven year-old children grouped by the body weight categories of underweight, normal healthy weight, overweight and obese revealed that dental decay decreased with increasing obesity. If one assumes that dental decay increases with sugar consumption, this observation suggests that increased sugar consumption may not be a primary cause of obesity.
